# Drivers and barriers to promoting self-care in individuals living with multiple long-term health conditions: a cross-sectional online survey of health and care professionals

**DOI:** 10.1186/s12889-025-21737-0

**Published:** 2025-03-05

**Authors:** Susan Barber, Benedict Hayhoe, Sonia Richardson, John Norton, Manisha Karki, Austen El-Osta

**Affiliations:** 1 Department of Primary Care & Public Health, Imperial College London, London, UK; 2 Self-Care Research Unit, Imperial College London School of Public Health, London, UK

**Keywords:** Self-care, Multimorbidity, COVID-19

## Abstract

**Background:**

Self-care is an important part of preventing unwarranted decline in poor health linked to multimorbidity and in maintaining or improving health. Health and social care professionals provide support for self-care, which can positively influence health and care outcomes. It is important to understand the extent to which they perceive their support to be effective and what barriers to its uptake and desired outcomes exist. This study investigated the knowledge, attitudes, and perceptions of front-line staff in England concerning drivers and barriers to promoting self-care in service users with multimorbidity.

**Methods:**

A cross-sectional online survey was administered via the Imperial College Qualtrics platform. Questions were asked about perceived drivers and barriers to promoting self-care in individuals with multimorbidity, including mental health. The quality of the survey was assessed by completing the Checklist for Reporting Results of internet E-Surveys (CHERRIES).

**Results:**

Extant barriers associated with service-users’ ability and opportunity for self-care identified by seventy H&SCPs in England were feelings of loneliness and social isolation (18.9%; *n* = 54) and mobility and access issues (14.3%; *n* = 41). The methods most commonly used to support self-care were social prescribing (18%; *n* = 55), helping service users monitor their symptoms (15.4%;*n* = 47), and referring to recognised programmes to support self-management (14.1%; *n* = 43). The factors most identified as positively affecting service users to self-care included knowledge and understanding about the benefits of self-care (92.5%; *n* = 62), support to improve limitations caused by a health condition (92.5% *n* = 62), and support to improve mental health and wellbeing (91% *n* = 61). Gaps in H&SCPs knowledge were reported, including how to improve practical interactions to sustain health seeking behaviours by service users (32.2%; *n* = 48), health coaching (21.5%; *n* = 32), and knowledge about effective self-care interventions (20.1%; *n* = 30). Most respondents (92.9%; *n* = 64) reported that the COVID-19 pandemic highlighted the need for self-care, and 44.9%(*n* = 31) agreed that the pandemic had a positive impact on their ability to promote self-care among service users.

**Conclusions:**

Self-care is important for service users who live with multimorbidity. H&SCPs identified loneliness, social isolation, mobility and access to services, and support in understanding or complying with a medical regimen as key obstacles to self-care for service users. Extant barriers identified by H&SCPs were associated with service users’ ability and opportunity to sustain self-care, especially relating to feelings of loneliness and experiences of social isolation, mobility and access issues.

**Supplementary Information:**

The online version contains supplementary material available at 10.1186/s12889-025-21737-0.

## Background

An increasing number of people are living with multimorbidity (MM), defined as the coexistence of two or more chronic health conditions in the same individual [1–3]. Service users with MM must cope with a complex array of associated issues, including the effects of the health conditions themselves, a greater treatment burden, polypharmacy, multiple health care appointments, psychosocial issues, and stress [4, 5].

Self-care may assist individuals with MM in meeting some of these needs. Among the positive outcomes of supported self-care for individuals living with MM are improved self-efficacy, independence in daily activities, quality of life, goal achievement [6], improved depression scores [7], diet [8], and composite measures of glycaemic control, blood pressure, lipids, and depression scores [7, 9, 10]. Of particular importance in the context of health services with limited resources is the potential for reduced use of health services, hospital admissions and emergency services [11–15] and prevention of illness progression [16].

Health and social care providers utilise a variety of interventions to support individuals with MM, including the deployment of strength-based approaches [17–21], the promotion of self-care, and self-management strategies [22]. Examples include access to local assets such as local community services, information and support, signposting and social prescribing, the use of shared decision-making, and alternative person-centred approaches to promote patient activation and self-care [17–19, 22].

Health and social care professionals (H&SCPs) who are well trained and organised are likely to support people and to respond better to the challenges of supporting people with MM to self-care [16]. There is a need to reform the way the healthcare system can deliver care, train, and reskill H&SCPs, with a focus on the development of necessary working relationships designed to support service users in self-care [23, 24]. Multiple barriers to supporting self-care among individuals with MM have been identified [25–28] such as complex psychosocial and cultural barriers and practicalities relating to roles and responsibilities among service users and health and social care providers [29].

### Study rationale

There is little comparative research available to guide our understanding of what the health and social care workforce considers to be drivers of and barriers to high-quality supported self-care for people who live with MM. Understanding how the self-care agenda is supported by H&SCPs is important. Although many studies have focused on the challenges and outcomes of self-care interventions [6–12, 14–16, 20, 21], to our knowledge, this is the first study to focus on the knowledge, attitudes and perceptions of H&SCPs about extant barriers. National guidance calls for H&SCPs to support self-care as part of personalised care across the health and care system [23, 30], while international guidance calls for personalised and supported self-care to be considered key to improving patient safety [2]. National guidance suggests that this requires a shift in relationships between H&SCPs and people, in which all the person’s health conditions and needs are considered together and in which that person’s own skills, strengths and attributes are considered [23]. Little is known about the additional needs of H&SCPs to help deliver this.

## Methods

### Aim, design and setting of the study

This study aimed to investigate the knowledge, attitudes, and perceptions (KAP) of H&SCPs in England concerning extant barriers and drivers to promote self-care among service users with multimorbidity.

We conducted a cross-sectional online survey of H&SCPs in England about their KAP, drivers and barriers to promoting self-care among service users. The survey was developed for this study, and is available as a supplementary file entitled “survey”. The setting of the study was the health and social care workforce.

The survey questions were developed based on a literature review and iterative feedback from the research team. While no formal qualitative work preceded survey development, items were carefully crafted to align with the study’s objectives. The questionnaire was pilot tested by five H&SCPs prior to the full launch to ensure its face validity, readability, and technical functionality. Feedback from the pilot testing informed minor revisions.

The recruitment process involved targeted invitations sent via professional networks, mailing lists, and social media platforms aimed at H&SCPs working in England. Although we did not directly ask respondents about their geographical location, the survey distribution methods ensured that the majority of respondents were likely from England.

The link to the electronic open survey was published and available on the Imperial College Qualtrics platform between 10th May 2021 and 18th August 2021; the survey could be accessed by anyone with a link. Potentially eligible participants received an invitation email, including a Participant Information Sheet and link to the survey from the study team; the first question in the survey requested informed consent from the participant after which the survey could be completed. Informed consent to participate was obtained from all of the participants in the study. Completion of the survey was voluntary. No incentives to complete the survey were offered. The data collected were stored in the Imperial College London Secure Database, and only the team researchers could access the survey results.

The survey comprised a total of 14 questions and was accessible using a personal computer or smartphone. The survey was visually optimized for these platforms, ensuring usability and a clean interface. The questions were displayed on one page with a progress bar. Some questions invited respondents to tick more than one response where that was appropriate, resulting in totals that may exceed 100%. Participants could review their answers before submitting them. Website cookies were used to assign a unique user identifier to each client’s computer. While IP addresses were collected temporarily during the data collection phase to prevent duplicate responses, no duplicate checks were performed in this study. IP data were deleted during the analysis phase to ensure complete anonymity. The technical functionality of the online survey was tested before it was published.

Drivers and barriers for the health and social care workforce to promote self-care in individuals living with MM were evaluated by establishing participants’ professional experiences of providing services. This was explored through questions estimating the proportion of service users with MM, their most common needs, and the methods used by respondents to promote self-care to manage service-user conditions. Questions on H&SCPs training were asked, as well as questions exploring perceived gaps in education. Some questions were multiple choice, and allowed respondents to choose as many potential responses as were relevant to answer the response. For example, to indicate all of the means by which the service user’s need for supported self-care was identified. A variety of question formats were used, including statements rated against Likert-style scales.

### Statistical analysis

Quantitative data were collected using an eSurvey questionnaire administered on Qualtrics. The survey responses were summarised using frequencies and percentages. All analyses were performed using SPSS (Statistical Package for Social Sciences) version 28.0.1. The quality of the survey was assessed by completing the Checklist for Reporting Results of internet E-Surveys (CHERRIES) [31].

## Results

A total of 70 participants completed the survey. Initially, 88 individuals clicked on the survey; 13 responses were excluded as they were recorded during a beta test version of the survey conducted prior to its official launch on May 10th, 1 respondent did not provide consent, and 4 respondents provided consent but did not answer any questions. This left 70 valid responses for analysis. Respondents could indicate more than one professional role, leading to a total of 79 role-related responses.

### Table 1: Characteristics of respondents

**Table 1:** Demographic profile of respondents

	*N*	(%)
**Age**		
20–29	10	(15.2)
30–39	10	(15.2)
40–49	14	(21.2)
50–59	28	(42.4)
60 & over	4	(6.1)
**Gender**		
Female	46	(68.7)
Male	21	(31.3)
**Ethnicity**		
White	52	(77.6)
Asian/Asian British	8	(11.9)
Black/Black British	3	(4.5)
Prefer not to say	2	(3.0)
Mixed	1	(1.5)
Other	1	(1.5)
**Occupation**		
Allied Health Care Professional	29	(36.7)
Other	15	(19.0)
GP	11	(13.9)
Nurse	7	(8.9)
Social care	4	(5.1)
Social prescriber	4	(5.1)
Pharmacist	4	(5.1)
Advocacy worker	2	(2.5)
Health coach	1	(1.3)
Rehabilitation	1	(1.3)
Hospital physician	1	(1.3)

The majority of participants were aged between 50 and 59 years (42.4%), followed by those aged 40–49 years (21.2%). Most respondents were female (68.7%), with males representing 31.3% of the sample. Ethnicity data revealed that 77.6% of respondents identified as White, with smaller proportions identifying as Asian/Asian British (11.9%), Black/Black British (4.5%), and other categories.

In terms of occupation, General Practitioners (GPs) accounted for 13.9% of respondents, while Allied Health Professionals (AHPs) made up the largest group at 36.7%. Other roles included social care professionals (5.1%), social prescribers (5.1%), pharmacists (5.1%), and nurses (8.9%). Additional roles such as advocacy workers, health coaches, rehabilitation specialists, and hospital physicians each comprised a small percentage of the sample.

**Table 2:** Survey findings

	*N*	(%)
**1. Need for self-care for individuals with multimorbidity most commonly identified in your service?**		
Direct referrals (e.g., GP, Out of Hospital, Mental health services)	52	(41.3)
Service-user self-referrals	25	(19.8)
Other	17	(13.5)
Drop-in service/signposting from community	13	(10.3)
Use a patient database/stratification according to a type of condition	12	(9.5)
Algorithm/segmentation to prioritise specific cohort(s) of service-users	5	(4.0)
Not applicable - my service is not concerned with identifying self-care needs	2	(1.6)
**2. Estimated proportion of service-users that have two or more long-term health conditions**		
0 to 25%	4	(6.0)
26 to 50%	11	(16.4)
51 to 75%	22	(32.8)
76 to 100%	30	(44.8)
**3. What are the most common needs of your service-users with multimorbidity?**		
Social isolation & loneliness	54	(18.9)
Access & mobility	41	(14.3)
Support to understand or comply with medical regimen	29	(10.1)
Computer/technical literacy	26	(9.1)
Caring responsibilities	24	(8.4)
Housing	22	(7.7)
Other	18	(6.3)
Support for end-of-life care	18	(6.3)
Need for advocate	15	(5.2)
Information about medicines	14	(4.9)
Need for interpreter/translator	13	(4.5)
The cost of items required to self-care	12	(4.2)
**4. To what extent do you feel that you/ the service is currently enabled to support users in the following?**		
**Promoting knowledge & health literacy**		
A Lot	32	(47.1)
A moderate amount	23	(33.8)
A little	13	(19.1)
**Improving mental wellbeing**		
A Lot	28	(40.6)
A moderate amount	32	(46.4)
A little	9	(13.0)
**Promoting physical activity**		
A Lot	35	(52.2)
A moderate amount	19	(28.4)
A little	13	(19.4)
**Improving healthy eating habits**		
A Lot	19	(27.9)
A moderate amount	21	(30.9)
A little	28	(41.2)
**Risk avoidance**		
A Lot	22	(32.4)
A moderate amount	28	(41.2)
A little	18	(26.5)
**Practising good hygiene**		
A Lot	20	(29.4)
A moderate amount	24	(35.3)
A little	24	(35.3)
**Rational and responsible use of products & services**		
A Lot	22	(32.4)
A moderate amount	24	(35.3)
A little	22	(32.4)
**5. Methods used to help promote self-care for people with multimorbidity**		
Social prescribing or signposting to suitable services	55	(18.0)
Helping patients to monitor their symptoms and know when to take action (including wearables & self-testing)	47	(15.4)
Recommend a recognised programme to support self-management	43	(14.1)
Provision of information about (please specify):	31	(10.1)
Motivational interviewing	31	(10.1)
Shared decision-making aid (e.g., Needs Assessment Tool)	29	(9.5)
IAPT or other community based mental health service	25	(8.2)
Peer to peer support (e.g. health buddy)	20	(6.5)
Other (please specify):	13	(4.2)
Recognised model of collaborative care planning (please specify):	12	(3.9)
**6. Which methods of communication do you use when supporting service-users to self-care?**		
Face-to-face	67	(28.9)
Telephone	60	(25.9)
Video chat	35	(15.1)
E-mail/Letter	32	(13.8)
SMS	27	(11.6)
Other:	11	(4.7)
**7. To what extent do you agree with the following statements?**		
**It is important to motivate service-users to self-care**		
Agree	67	(95.7)
Neutral	2	(2.9)
Disagree	1	(1.4)
**Self-care is the responsibility of service-users themselves**		
Agree	39	(55.7)
Neutral	23	(32.9)
Disagree	8	(11.4)
**It is the responsibility of the healthcare professionals to promote self-care**		
Agree	65	(92.8)
Neutral	4	(5.7)
Disagree	1	(1.4)
**The service-user’s level of self-confidence is an important factor in determining the extent to which they self-care effectively**		
Agree	64	(91.4)
Neutral	6	(8.6)
Disagree	0	(0)
**I/ my organisation can influence the service-user’s capabilities to self-care**		
Agree	60	(85.8)
Neutral	8	(11.4)
Disagree	2	(2.8)
**I am able to influence the self-care capability of my service-users**		
Agree	59	(84.3)
Neutral	9	(12.9)
Disagree	2	(2.9)
**Service-users are more likely to make changes to their lifestyle & behaviours as a result of the support provided by my service**		
Agree	57	(82.6)
Neutral	9	(13.0)
Disagree	3	(4.3)
**I take into account the service-user’s personal circumstances when considering the type or level of support I provide to them**		
Agree	68	(97.1)
Neutral	2	(2.9)
Disagree	0	0
**Inequalities can have a detrimental effect on people’s ability to self-care**		
Agree	69	(98.6)
Neutral	0	(0)
Disagree	1	(1.4)
**8. In your experience**,** which of the following negatively affect service-users’ ability to self-care?**		
**Lack of knowledge about self-care activities**		
Agree	61	(91.0)
Neutral	4	(6.0)
Disagree	2	(3.0)
**Time available to self-care**		
Agree	46	(68.7)
Neutral	8	(11.9)
Disagree	13	(19.4)
**Lack of positive feedback**		
Agree	55	(82.1)
Neutral	6	(9.0)
Disagree	6	(9.0)
**Low motivation**		
Agree	63	(94.0)
Neutral	2	(3.0)
Disagree	2	(3.0)
**Low empowerment**		
Agree	59	(88.1)
Neutral	6	(9.0)
Disagree	2	(3.0)
**Costs (e.g.**,** wearables**,** gym subscription)**		
Agree	48	(71.6)
Neutral	12	(17.9)
Disagree	7	(10.4)
**Lack of support from healthcare professionals**		
Agree	48	(71.6)
Neutral	13	(19.4)
Disagree	6	(9.0)
**Lack of support from family/carers**		
Agree	49	(73.1)
Neutral	13	(19.4)
Disagree	5	(7.5)
**Lack of support from community mental health team**		
Agree	44	(65.7)
Neutral	19	(28.4)
Disagree	4	(6.0)
**9. Which factors do you believe positively affect service-users to self-care?**		
**Good understanding of benefits of self-care**		
Agree	62	(92.5)
Neutral	2	(3.0)
Disagree	3	(4.5)
**Desire to prevent non-communicable diseases**		
Agree	41	(61.2)
Neutral	19	(28.4)
Disagree	7	(10.4)
**Access to remote monitoring & other technology**		
Agree	43	(64.2)
Neutral	19	(28.4)
Disagree	5	(7.5)
**Support to help improve mental health & wellbeing**		
Agree	61	(91.0)
Neutral	3	(4.5)
Disagree	3	(4.5)
**Social prescribing support (e.g.**,** housing etc.**,**)**		
Agree	60	(89.6)
Neutral	5	(7.5)
Disagree	2	(3.0)
**Knowledge about the purposes of the medicines prescribed**		
Agree	56	(84.8)
Neutral	8	(12.1)
Disagree	2	(3.0)
**Support to help improve lifestyle limitations caused by a health condition**		
Agree	62	(93.9)
Neutral	1	(1.5)
Disagree	3	(4.5)
**10. Continuing Professional Development (CPD) is important to help me provide better self-care support**.		
Agree	59	(85.5)
Neutral	10	(14.5)
Disagree	0	(0)
**11. Have you participated in any education or training**,** or have you been mentored to develop your knowledge & skills in relation to the following?**		
Person-centred care	47	(25.3)
Advanced communication for shared decision making	35	(18.8)
Self-care	31	(16.7)
Personalised care	28	(15.1)
Social prescribing	20	(10.8)
Lifestyle medicine	16	(8.6)
Other	9	(4.8)
**12. Have you identified any gaps in your own knowledge that could help you promote self-care in service-users?**		
Practicalities/ sustaining health-seeking behaviours	48	(32.2)
Empowerment/ coaching service-users	32	(21.5)
Knowledge about effective self-care interventions	30	(20.1)
How to improve compliance/ adherence to medical regimen	29	(19.5)
Other	7	(4.7)
None	3	(2.0)
**13. The current pandemic has had a positive effect on my ability to promote self-care with service-users**.		
Agree	31	(44.9)
Neutral	25	(36.2)
Disagree	13	(18.8)
**14. The COVID-19 pandemic has highlighted the importance of supporting service-users to self-care**		
Agree	65	(92.9)
Neutral	5	(7.1)
Disagree	0	(0)

*Note*: The percentages in the table are calculated based on the number of respondents who answered each question (N = [specific sample size for each question]). Some questions allowed for multiple responses, which is reflected in the totals exceeding 100%

Not all participants responded to every question. The Table indicates the total number of respondents to each question, the total number of responses to each question and, where the question enabled multiple answers, the number of cases, or times, that a specific response was chosen. Responses to every question do not add up to 70/100%.

### Service-user identification and most common needs

More than half (77.6%; *n* = 52) of H&SCPs estimated that their patients had MM, defined as two or more long-term health conditions. The respondents indicated that many service users (41.3%; *n* = 52) were referred directly to the service, while 19.8% (*n* = 25) engaged with the provider following self-referral.

The needs that were most strongly associated with service users with MM were loneliness and social isolation (18.9%; *n* = 54), mobility and access issues (14.3%; *n* = 41), and support in understanding or complying with a medical regimen (10.1%; *n* = 29).

### Extent to which health and care professionals feel enabled to support self-care

Most respondents felt that they and/or that their organisations were able to support service users “a lot” (47.1%; *n* = 32) or a moderate amount (33.8%; *n* = 23) in improving health literacy levels. The respondents also felt that they were able to improve service users’ mental wellbeing (40.6%; *n* = 28 by a lot and 46.4%; *n* = 32 by a moderate amount), promote physical activity (52.2%; *n* = 35 by a lot, and 28.4%; *n* = 19 by a moderate amount), improve healthy eating habits (27.9%; *n* = 19 by a lot, and 30.9%; *n* = 21 a moderate amount), avoid risks (32.4%; *n* = 22 by a lot, and 41.2%; *n* = 28 to a moderate extent), improve hygiene practices (29.4%; *n* = 20 by a lot, and 35.3%; *n* = 24 a moderate amount), and promote the rational use of products and services 32.4%; *n* = 22 a lot, 35.3% (*n* = 24) a moderate amount.

### Methods used to promote self-care

Respondents signposted service-users to other services, including social prescribing (18%; *n* = 55), information services, including digital health technologies (e.g., wearables) and decision support tools (e.g., self-testing), to help individuals monitor their own symptoms (15.4%; *n* = 47) and other recognised programmes to support self-management (14.1%; *n* = 43).

### Methods of communication used to support self-care

Over half of the service providers reported that they mostly communicated with service users either face to face (28.9%; *n* = 67) or by telephone (25.9%; *n* = 60), with fewer communicating by video chat (15.1% *n* = 35), email or letter (13.8% *n* = 32), SMS (11.6% *n* = 27), or other means of communication (4.7% *n* = 11).

### Factors affecting service users’ ability to self-care

Factors most cited by H&SCPs that were perceived to positively affect service users’ ability to self-care included support to help improve lifestyle limitations caused by a health condition (93%; *n* = 62), a good understanding of the benefits of self-care (93%; *n* = 62), support to improve mental health and wellbeing (91%; *n* = 61) and receiving support via services such as social prescribing and to address unmet social needs, for example, housing (89.6%; *n* = 60).

The factors most cited by H&SCPs that were perceived to negatively impact service users’ ability to self-care included low motivation (94%, *n* = 63), lack of knowledge about self-care activities (91%, *n* = 61), and low empowerment (88.1%, *n* = 59). Additionally, 82.1% (*n* = 55) indicated that a lack of positive feedback had a negative impact on service users’ ability to self-care. Many respondents highlighted a lack of support from family/carers (73.1%; *n* = 49) and healthcare professionals (71.6% *n* = 48); the costs associated with self-care, such as gym membership or wearable aid (71.6%; *n* = 48); the time needed for self-care (68.7%; *n* = 46); and a lack of support from a community mental health team (65.7%; *n* = 44).

### Perceptions of responsibility and influence on the behaviour of service users

Most respondents agreed with the following statements: it is important to motivate service users to self-care (95.7%; *n* = 69); it is the responsibility of H&SCPs or their organisations to promote self-care (92.8%; *n* = 65) and (84.3%; *n* = 59), respectively. Many reported being able to positively influence the self-care capability of service users (85.8%; *n* = 60), and their perception was that service users are more likely to make changes to their lifestyle and behaviours because of the support provided (82.6%, *n* = 57). H&SCPs take into account service users’ personal circumstances when considering the type or level of support offered (97.1%; *n* = 68).

Most respondents agreed with the statement that inequalities can have a detrimental effect on people’s ability to self-care (98.6%; *n* = 69). Smaller numbers of responses were “neutral” or “disagreed” with the statements.

### Impact of the COVID-19 pandemic

Almost all respondents (92.9%; *n* = 64) felt that the pandemic highlighted the importance of supporting service users to self-care, and nearly half (44.9%; *n* = 31) felt that the pandemic had a positive effect on their ability to promote self-care among service users.

### Continuing professional development, education and training

Many respondents (85.5%; *n* = 59) agreed with the statement that continuing professional development was important to help provide better self-care support. One-quarter of the respondents (25.3%; *n* = 47) had participated in education and/or training to support person-centred care, and 18.8% (*n* = 35) had participated in training to support shared decision making.

Many respondents identified gaps in their knowledge, especially in relation to improving their understanding and skills to support service users in sustaining health-seeking behaviour (32.2%; *n* = 48) or coaching service users in self-care (21.5%; *n* = 32).

## Discussion

### Summary of principal findings

H&SCPs identified loneliness, social isolation, mobility and access to services, and support in understanding or complying with a medical regimen as key obstacles to self-care for service users. Extant barriers identified by H&SCPs were associated with service users’ ability and opportunity to sustain self-care, especially relating to feelings of loneliness and experiences of social isolation, mobility and access issues. There is agreement among researchers that social inequalities negatively impact health. For example, low income, inadequate housing, social class status, participation in social activities affect a person’s health [32, 33] and their opportunity to participate in activities designed to support self-management of long-term health conditions [34].

Service users’ knowledge and understanding are critical factors for successfully adopting self-care practices. Positive influences on self-care efforts and outcomes included support from family, carers, or professional services. In contrast, barriers such as low motivation, insufficient knowledge, lack of encouragement, and inadequate support, in addition to the costs and time involved, hinder the routine uptake of self-care behaviours outside clinical environments.

H&SCPs identified their strengths in employing person-centred methods to boost health literacy, mental health, hygiene, physical activity, and the rational use of health products and services among service users. However, fewer believed that their interactions significantly influenced long-term healthy eating habits.

Continued professional development was deemed crucial, and while many H&SCPs have received training on supporting self-care, gaps remain, particularly in strategies for practical engagement and maintaining and sustaining health-seeking behaviours, such as medication adherence and the need for more evidence-based and coaching-style methods to enhance self-care skills.

### Interpretation and comparison with the literature

The COVID-19 pandemic drew attention to the importance of self-care, with approximately half of the participants noting that the pandemic-induced care model improved their support for self-care. A recent study suggested that the pandemic altered H&SCPs’ attitudes toward self-care, advocating for its inclusion in healthcare education to sustain attitude shifts and boost individual empowerment [35].

Earlier studies on self-care provided useful information and detailed positive outcomes from interventions to improve self-care related to long-term conditions such as type 2 diabetes [36] and specific comorbidities such as cardiovascular disease and stroke [37], depression, diabetes, and heart disease [7]. These studies tend to focus on patient outcomes rather than KAP of H&SCPs. Thus, in the present study, H&SCPs reported the potential to positively influence the lifestyle and behaviour of service users in relation to certain behaviours, including exercise and good hygiene. Less certainty was reported regarding the extent to which lifestyle advice could help improve healthy eating habits. This finding contrasts with studies of supported self-management that reported significant positive outcomes for people with MM in relation to diet [8]. This may be attributed to the small number of respondents who were dieticians.

Social isolation, loneliness, a perceived lack of support to address lifestyle factors such as low motivation, poor knowledge about self-care activities, time constraints and associated costs could negatively impact an individual’s ability to self-care. H&SCPs indicated the potential detrimental impact of inequalities on service users with MM, posing additional challenges for both the person with MM and the service provider. Our findings are consistent with the results of a recent study that considered the impact of inequalities on the outcomes of self-care interventions in eight primary care locations among individuals with MM in Canada [34]. Of the six lifestyle factors under consideration, the likelihood of improvement was no longer significant for people with lower incomes and with lower educational status in the domains of emotional wellbeing, self-monitoring and insight [34].

The study reported above confirms that knowledge, training and preparedness on the part of H&SCPs are vital to supporting self-care [16]. Other studies highlighted the need for training among primary care staff to codesign supported self-care plans with clinical and patient input [38], for disease-specific training alongside training to support self-care [11, 39], and for better planned integrated care and training to improve multidisciplinary teamwork [40, 41]. The results of our study reported above drew attention to gaps in H&SCP knowledge about strategies for practical engagement and skills for maintaining health-seeking behaviours, such as medication adherence, and the need for more evidence-based and coaching-style methods to enhance self-care skills.

### Implications for clinicians and policy makers

Supported self-care is part of the NHS’s commitment to make personalised care business as usual across the health and care system [23, 30]. Supported self-care interventions are most successful if they are delivered as part of integrated care interventions for people living with long-term health conditions [42]. This highlights the need for a shift in the relationship between H&SCPs and service users, in which the health conditions and needs of the whole person are considered together [43] and in which that person’s own skills, strengths and attributes are considered [44]. Guidelines on supporting self-care are designed to support service providers in caring for people with long-term health conditions and start from the premise that people want to have choice and control over the way their care is planned and delivered, based on what matters to them and their individual strengths, needs and preferences [23].

The H&SCPs in our study reported that continuing professional development was important to them and that some had relevant training experience. However, the number that had completed training was small, and gaps in knowledge about how to improve practical interactions to sustain health-seeking behaviours were reported. This suggests that H&SCPs require more training, training opportunities, or experience to help them promote the uptake and sustained adoption of self-care among service users. In the UK, the NHS Learn and the Personalised Care Institute aim to address this gap by helping H&SCPs develop the knowledge and skills to support the delivery of a comprehensive model for personalised care, which, as a domain, has a direct bearing on self-care [45].

Additional training, learning on the job and mentoring may improve the knowledge and skills base of H&SCPs. This could be linked to the development of clearer clinical guidelines [46, 47], teamwork, and developing a broader consensus on the key elements of self-care likely to bring positive changes [11, 16, 38, 39, 48]. There is the potential to achieve this, for example, by bringing resources from services across the public sector to shape and reduce the negative impacts of the social determinants of health [49].

### Strengths and limitations

To our knowledge, this is the first study in the UK that sought to investigate the KAP of H&SCPs in the UK concerning extant barriers and drivers to promote self-care among service users with MM.

A key strength of our study is that it provided insights from a variety of service providers about what barriers continue to be challenging to individuals with MM and to the health and care professionals themselves. To our knowledge, only one other study has sought to investigate the prevailing attitudes of H&SCPs toward self-care following the advent of the COVID-19 pandemic [35], and it considered a general population of service users, while our study specifically focused on a cohort of service users who live with MM. The main limitation of our study was that it collected data from just 70 respondents, and for this reason, we cannot assume that the cross-sample is representative of the health and care workforce. Our data broadly reflects similar percentages of women and men and similar ethnic backgrounds to the NHS workforce [50, 51]. Due to the nature of the online survey, obtaining a response rate was not possible. However, the results demonstrate that the study attracted a broad representation of roles from a range of H&SCPs, offering a comprehensive view of the systemic challenges and facilitators in promoting self-care in the context of MM.

Understanding the needs of individuals who have MM and the needs of H&SCPs who care for them is important. Our study asked H&SCPs about the extent to which they were able to meet service users’ needs, the type of training they had completed and outstanding gaps in their training and experience. In addition, it elicited their perceptions about the role of service users and the factors that impact service users’ ability to self-care. This is important in the current policy and service provision context in which the agency of individuals with MM must be taken into account in supporting the development and use of person-centred/personalised care models [19, 26]. The study highlighted gaps in knowledge among service providers, which, if addressed, would improve their ability to support people with MM and would likely improve self-care and potential health outcomes.

### Outstanding questions/implications for research

More research is required to better understand the types of training or other methods of skill and knowledge acquisition by H&SCPs, which could improve the support available to people living with MM. Future research could usefully focus on what might equip H&SCPs to become more knowledgeable and confident in choosing strategies and interventions designed to improve motivation and meet service-users’ needs.

The strategies and techniques most frequently used by respondents in our survey were social prescribing, helping service users monitor their symptoms and knowing when to act, with a majority indicating the importance of service-user motivation in behaviour change. Social prescribing was designed to address challenges and put person-centred care centre stage. Two systematic literature reviews reported mixed results. Both reported some improvements across a range of outcomes, and limited exploration of the impact of social prescribing. One reported on some limitations of social prescribing as an effective approach to improving health outcomes for adults experiencing MM and social deprivation in primary and community care settings [52]. Both found that the quality of the evidence remains poor [52, 53].

Further research could explore the drivers of and barriers to optimal outcomes resulting from social prescribing by H&SCPs and service users.

Additionally, more research into the best use of communication channels may be useful. Our study suggested that communication with service users was mostly face-to-face and by telephone, but respondents also reported the use of newer communication tools, e.g., video chat. New research could explore the extent to which H&SCPs can influence service users’ ability to self-care if delivered remotely and support person-centred and proactive care.

## Conclusions

MM is increasingly becoming a global concern, primarily due to the aging population and lifestyle changes. Knowledge and understanding on the part of service users with MM is key to successful engagement with and acceptance of evidence-based self-management and self-care interventions. Policymakers should therefore focus on ensuring that appropriate information, training, mentoring and support is available to patients and H&SCPs. The pandemic shed light on the important role that self-care could play in patients with MM. Changed models of care may result in improvements in H&SCPs’ ability to support service users in their personal self-management and self-care journeys. More research is needed to understand changes that may be due to the pandemic and its impact and how any lessons can be applied to promote the sustained adoption of health-seeking self-care behaviours.

## Electronic supplementary material

Below is the link to the electronic supplementary material.

Supplementary Material 1

## Data Availability

The dataset used and analysed during the current study is available within the paper in Tables 1 and 2. The survey instrument is available. See supplementary material.

## Declarations

### Patient and public involvement

Co-author’s Sonia Richardson and and John Norton were public partners of Imperial College, School of Public Health, The National Institute for Health Research Applied Research Collaboration for Northwest London. Both were lay researchers and brought their direct and indirect experience of living with and caring for others who had multimorbidity to the development and analysis of study data.

## Abbreviations

MM: Multimorbidity
H&SCPs: Health and Social Care Professionals
KAP: Knowledge attitudes and perceptions

## Glossary

**Health and social care professionals**: Health and social care professionals work for the NHS in England and for Social Services in England. The term covers a wide range of professionals who care for people living with more than one long-term health condition/multimorbidity. Examples include general practitioners, specialist consultants, physiotherapists, dieticians, nurses, social workers.
**Social prescriber**: Social prescriber’s can be employed by NHS and Social Services. A key role is to support people with long term health conditions to identify and utilise available services within and outside of the NHS and Social Services to support self-care.
**Social prescribing**: Social prescribers help people living with long term health conditions to understand what services are available to them that can support them to self-care. Social prescribers are knowledgeable about what local community services are available within a relevant geography that could enhance the quality of life, and of support experienced by the individual. For example, exercise classes, walking groups, local carers associations, sources of information about their health conditions and how best to self-care.
**Health literacy**: Health literacy refers to a person’s knowledge about health issues. For people who live with multiple long term health conditions it may be crucial for them to recognise symptoms, understand when it is important to act, and what sort of action to take, understand the importance of taking care of themselves and how to do so. This may relate to a wide range of practices, for example, taking medicines as prescribed or otherwise being able to build the necessary skills to look after their health.
**Wearables**: In this paper, we refer to wearables to aid self-care and/or to provide feedback to health care professionals. Examples of wearables include heart rate monitors, fitness trackers, insulin sensors.
