# Supplementary material for: Drivers and barriers to promoting self-care in individuals living with multiple long-term health conditions: a cross-sectional online survey of health and care professionals
Source: BMC Public Health. 2025 Mar 5;25:884. doi: 10.1186/s12889-025-21737-0 (PMC11883917; doi:10.1186/s12889-025-21737-0)
Supplement: Supplementary file 1 — Supplementary Material 1 [file 12889_2025_21737_MOESM1_ESM.pdf]

(SMS) Self-Care for Multimorbidity Study

Start of Block: Default Question Block

Q1 Providers of health and social care are using new models of support for people who live with multimorbidity, frailty & mental health conditions, including the deployment of strength-based approaches & the promotion of self-care strategies. Examples include access to local assets, information and support, signposting, social prescribing, use of shared decision making and alternative person-centred 'lifestyle medicine' approaches to promote patient activation and self-care. We're keen to hear your views & experiences on what health & social care workforce staff consider as drivers & barriers to good quality supported self-care. Thank you for participating in this short (10 min) survey. Please refer to our Participant Information Sheet or more information.

Q2 Do you consent to take part in this survey? If yes, you understand that: Your participation is voluntary & you are free to withdraw at any time, without giving any reason and without your legal rights being affected. Your data will be stored securely on an encrypted & secure institutional platform. Information collected may be used to support other research in the future. I give permission for Imperial College London to access my records that are relevant to this research.

○ Yes (1)

○ No (2)

Q3 What occupational background best describes you?

- ☐ GP (1)
- ☐ Nurse (2)
- ☐ Allied Health Care Professional (3)
- ☐ Health coach (9)
- ☐ Social care (10)
- ☐ Rehabilitation (8)
- ☐ Social prescriber (7)
- ☐ Advocacy worker (12)
- ☐ Psychiatrist (13)
- ☐ Mental health nurse (14)
- ☐ Peer worker (15)
- ☐ Health care assistant (21)
- ☐ Occupational therapy (16)
- ☐ Mental health recovery (17)
- ☐ Pharmacist (18)
- ☐ Hospital physician (19)
- ☐ Hospital nurse (20)

☐ Other (Please state which) (11) ____

Q4 How is the need for self-care for individuals with multimorbidity most commonly identified in your service? Tick all that apply.

☐ Direct referrals (eg. GP, Out Of Hospital, Mental health services) (6)

☐ Use a patient database / stratification according to type of condition (1)

☐ Algorithm / segmentation to prioritise specific cohort(s) of service users (2)

☐ Drop-in service / signposting from community (3)

☐ Service user self-referrals (4)

☐ Not applicable - my service is not concerned with identifying self-care needs (7)

☐ Other (please specify): (5) ____

Q5 Please estimate what proportion of the service users that you care for have two or more long-term health conditions?

	0 10 20 30 40 50 60 70 80 90 100
% with two or more long-term health conditions ()	

Q6 What are the most common needs for your service users with multimorbidity? Please select all that apply.

- ☐ Computer/technical literacy (2)
- ☐ Support to understand or comply with medical regimen (10)
- ☐ Housing (3)
- ☐ Caring responsibilities (4)
- ☐ Social isolation & loneliness (5)
- ☐ The cost of items required to self-care (6)
- ☐ Access & mobility (11)
- ☐ Need for advocate (8)
- ☐ Need for interpreter/ translator (14)
- ☐ Information about medicines (17)
- ☐ Support for end of life care (18)
- ☐ Other (please specify): (13)

____

Q7 There are 7 Pillars of Self-Care which describe the activities that individuals can practice. To what extent do you feel that YOU/ THE SERVICE is currently enabled to support users in adopting health-seeking self-care practices?

	(1) None at all (44)	(2) A little (45)	(3) A moderate amount (46)	(4) A lot (47)	(5) A great deal (48)
(1) Promoting knowledge & health literacy (1)	○	○	○	○	○
(2) Improving mental wellbeing (28)	○	○	○	○	○
(3) Promoting physical activity (29)	○	○	○	○	○
(4) Improving healthy eating (30)	○	○	○	○	○
(5) Risk avoidance (31)	○	○	○	○	○
(6) Practicing good hygiene (32)	○	○	○	○	○
(7) Rational and responsible use of products & services (33)	○	○	○	○	○

Q8 What methods do you use to help promote self-care for people with multimorbidity? Please indicate all that apply.

- ▢ Recommend a recognised programme to support self-management (2)
- ▢ Shared decision-making aid (e.g., Needs Assessment Tool) (3)
- ▢ Helping patients to monitor their symptoms and know when to take action (including wearables & self-testing) (5)
- ▢ Social prescribing or signposting to suitable services (6)
- ▢ Motivational interviewing (16)
- ▢ Peer to peer support (e.g. health buddy) (9)
- ▢ IAPT or other community based mental health service (17)
- ▢ Provision of information about (please specify): (14) ____
- ▢ Recognised model of collaborative care planning (please specify): (15) ____
- ▢ Other (please specify): (11) ____

Q9 Which methods of communication do you use when supporting service users to self-care? Please indicate all that apply.

- ☐ Face-to-face (1)
- ☐ Telephone (2)
- ☐ SMS (3)
- ☐ E-mail/ Letter (5)
- ☐ Video chat (6)
- ☐ Other (please specify): (4) ____

Q10

To what extent do you agree with the following statements?

	Strongly Disagree (1)	Disagree (2)	Neutral (3)	Agree (4)	Strongly Agree (5)
It is important to motivate service users to self-care (4)	○	○	○	○	○
Self-care is the responsibility of service users themselves (9)	○	○	○	○	○
It is the responsibility of the healthcare professionals to promote self-care (10)	○	○	○	○	○
The service user's level of self confidence is an important factor in determining the extent to which they self-care effectively (7)	○	○	○	○	○
I/ my organisation can influence the service user's capabilities to self-care (1)	○	○	○	○	○
I am able to influence the self-care capability of my service	○	○	○	○	○

users (3)					
Service users are more likely to make changes to their lifestyle & behaviours as a result of the support provided by my service (2)					
I take into account the service user's personal circumstances when considering the type or level of the support I provide to them (8)					
Inequalities can have a detrimental effect on people's ability to self-care (11)					
The COVID-19 pandemic has highlighted the importance of supporting service users to self-care (6)					
The current pandemic has had a positive effect on my ability to promote self-care with					

service users (12)					
Continuing Professional Development (CPD) is important to help me provide better self-care support (5)	○	○	○	○	○

Q11 Have you participated in any education or training, or have you been mentored to develop your knowledge & skills in relation to the following:

- ☐ Lifestyle medicine (1)
- ☐ Self-care (7)
- ☐ Personalised care (8)
- ☐ Person centred care (2)
- ☐ Social prescribing (3)
- ☐ Advanced communication for shared decision making (4)
- ☐ None of the above (9)
- ☐ Other (please state) (6) ____

Q12 Have you identified any gaps in your own knowledge that could help you promote self-care in service users ?

- ☐ Knowledge about effective self-care interventions (1)
- ☐ How to improve compliance/ adherence to medical regimen (4)
- ☐ Empowerment/ coaching service users (5)
- ☐ Practicalities/ sustaining health seeking behaviours (6)
- ☐ Other (please specify) (7)
- ☐ None (8)

Skip To: Q14 If Have you identified any gaps in your own knowledge that could help you promote self-care in servi... = None

Carry Forward Selected Choices from "Have you identified any gaps in your own knowledge that could help you promote self-care in service users ? "

Q13 Please rank which areas of knowledge of self-care you lack, or that you would like to improve:
______ Knowledge about effective self-care interventions (1)
______ How to improve compliance/ adherence to medical regimen (2)
______ Empowerment/ coaching service users (3)
______ Practicalities/ sustaining health seeking behaviours (4)
______ Other (please specify) (5)
______ None (6)

Q14 In your experience, which of the following **negatively** affect service users ability to self-care?

	Strongly agree (18)	Somewhat agree (19)	Neutral (20)	Somewhat disagree (21)	Strongly disagree (22)
Lack of knowledge about self-care activities (17)	○	○	○	○	○
Time available to self-care (11)	○	○	○	○	○
Lack of positive feedback (25)	○	○	○	○	○
Low motivation (12)	○	○	○	○	○
Low empowerment (26)	○	○	○	○	○
Costs (e.g. wearables, gym subscription) (13)	○	○	○	○	○
Lack of support from healthcare professional (22)	○	○	○	○	○
Lack of support from family/carers (23)	○	○	○	○	○
Lack of support from community mental health team (27)	○	○	○	○	○
Other (please state) (15)	○	○	○	○	○

Q15 Which factors do you believe **positively** affect service users to self-care?

	Strongly agree (4)	Somewhat agree (5)	Neutral (6)	Somewhat disagree (7)	Strongly disagree (8)
Good understanding of benefits of self-care (10)	o	o	o	o	o
Desire to prevent non communicable diseases (8)	o	o	o	o	o
Access to remote monitoring & other technology (7)	o	o	o	o	o
Support to help improve mental health & wellbeing (12)	o	o	o	o	o
Knowledge about the purposes of the medicines prescribed (15)	o	o	o	o	o
Social prescribing support (e.g. housing etc.,) (13)	o	o	o	o	o
Support to help improve lifestyle limitations caused by a health condition (14)	o	o	o	o	o
Other (please state) (9)	o	o	o	o	o

Q16 What is the single most important thing that you feel you could do to promote self-care among your service users?

____

Q17 How old are you?

- ◯ Years: (1) ____

Q18 What is your gender?

- ◯ Male (1)
- ◯ Female (2)
- ◯ Other (3)
- ◯ Would rather not say (4)

Q19 What is your ethnicity?

- ◯ White (1)
- ◯ Black/Black British (2)
- ◯ Asian/Asian British (3)
- ◯ Mixed (4)
- ◯ Other (please specify): (5) ____
- ◯ Prefer not to say (6)

Q20
Thank you for taking the time to respond.
We are looking to interview up to 30 participants (via telephone, Skype or Microsoft Teams) to gain a deeper understanding of this topic. If you are interested in being contacted for an interview (approximately 30 minutes), please enter contact information and we will be in touch; if you are not interested please click next to register your responses. All information will be confidential.

- ○ Name: (1) ____
- ○ Email: (2) ____
- ○ Phone number: (3) ____

End of Block: Default Question Block
